# Preventing Stunting in South African Children Under 5: Evaluating the Combined Impacts of Maternal Characteristics and Low Socioeconomic Conditions

**DOI:** 10.1007/s10935-024-00766-2

**Published:** 2024-02-28

**Authors:** Handan Wand, Sarita Naidoo, Vaneshree Govender, Tarylee Reddy, Jayajothi Moodley

**Affiliations:** 1 Biostatistics and Databases Program, Kirby Institute, University of New South Wales (UNSW Sydney), Kirby Institute Level 6, Wallace Wurth Building, Kensington, NSW 2052 Australia; 2 Numolux Group, Pretoria, South Africa; 3 The Aurum Institute, Johannesburg, South Africa; 4 Biostatistics Unit, South African Medical Research Council, Durban, Kwazulu-Natal South Africa

**Keywords:** Stunting, South Africa, Children, Population-level evaluation

## Abstract

More than 140 million children under five suffered from stunting in 2020. This highlights the ongoing challenge of addressing childhood malnutrition globally. We utilized data from a nationally representative sample of children under five years of age (n = 14,151) who participated in five cycles of the South African National Income Dynamics Study (SA-NIDS) (2008–2017). We estimated the proportion of stunted children attributed to the mothers’ anthropometric characteristics and socioeconomic conditions. We also quantified the population-level burden of low-socioeconomic conditions on hunger/food insecurity among pregnant women (n = 22,814) who participated in the nine rounds of the South African General Household Surveys (GHS) (2008–2021). Results from weighted-multivariate logistic regression were incorporated into the population-level impacts of correlates of stunting and low-socioeconomic conditions. The prevalence of stunting declined from 25% in 2008 to 23% in 2017. Mothers’ anthropometric measures (underweight/height < 160 cm), marital status, low education, absence of medical insurance and low-socioeconomic conditions were all identified as the most influential risk factors for stunting. Their population-level impacts on stunting increased substantially from 34% (in 2008) to 65% (in 2017). Comprehensive strategies emphasizing enhanced food security, extended breastfeeding, appropriate nutrition, and access to adequate healthcare and education are urgently needed to reduce the burden of food insecurity low-socioeconomic, malnutrition, and its long-term consequences.

**Supplementary Information:**

The online version contains supplementary material available at 10.1007/s10935-024-00766-2.

## Introduction

Severe poverty is one of the major drivers of malnutrition, as it limits access to nutritious food, clean water, and adequate healthcare (World Health Organization (WHO) & United Nations Children’s Fund (UNICEF), 2019). Child malnutrition which can profoundly affect a child’s growth and development, is a major global health concern with significant consequences (WHO, 2020). Stunting, a severe form of growth failure, is characterized by low height-for-age, indicating a child has not reached the expected height for their age (WHO Child Growth Standards, 2007). Chronic child malnutrition is one of the leading causes of stunting in children, which can be irreversible. Stunting can have short- and long-term consequences, including developmental delays, poor cognitive function, and increased risk of chronic diseases such as diabetes and heart disease. Stunted adults often exhibit reduced economic productivity, which can perpetuate the cycle of poverty and malnutrition (Black et al., 2013). The disparities in stunting rates between and within countries highlight the significant burden of malnutrition and its complex relationship with poverty (Alderman et al., 2006; Hoddinott et al., 2013; Martins et al., 2011).

Despite efforts to reduce childhood malnutrition and its severe consequences remain a significant global health challenge, with more than 140 million children under five years of age affected in 2020 (WHO Malnutrition, 2019). Stunting, in particular, is a major public health concern in many low- and middle-income nations, especially in Sub-Saharan countries, including South Africa. The country has one of the world’s highest stunting rates, with one in four children facing stunted growth due to malnutrition (Kaldenbach et al., 2022).

One of the targets included in the WHO 2012–2025 global action plan is to achieve a 40% reduction of stunted children under-5 by 2025 (WHO Global Action, 2013; De Onis et al., 2013). Aligned with these targets, awareness and prevention programs were developed to emphasize the public health importance of maternal and child malnutrition. Many non-governmental organizations work in partnership with governments and local communities to implement nutrition-specific and -sensitive interventions. These programs particularly focussed on implementing community-based initiatives to promote a healthy and balanced diet worldwide. In addition to emphasizing the importance of exclusive breastfeeding for the first six months, mothers were also provided with the necessary micronutrients and educated on hygiene and nutritional food preparation (Bahl et al., 2005; Black et al., 2013; Horta et al., 2015; Kaldenbach et al., 2022). Given the intense efforts made to reduce maternal and child malnutrition over the past decade, assessing their impact on growth deficiencies in children is crucial.

Using data from multiple rounds of national surveys conducted in South Africa, this study examined the temporal trends of stunting in children under five. Additionally, we assessed the population-level effects of socioeconomic factors, maternal attributes, and their interactions with stunting. Given the adverse effect of severe malnutrition on maternal health, we also quantified the burden of low socioeconomic conditions on food insecurity among pregnant women who participated in General Household Surveys (2008–2021). This aspect of our analysis provided a unique opportunity to understand the impact of the COVID-19 pandemic on food insecurity, particularly in comparison to the pre-pandemic period. Although the long-term implications of the pandemic on child malnutrition and stunting are yet to be fully assessed, it is crucial to monitor and address these challenges as they emerge. This study provides detailed insights into the low-socioeconomic conditions and their contributions to stunting in South African children under five. Although numerous studies have identified the correlates and predictors of stunting across diverse populations, this is the first study to quantify their population-level burden in South Africa, which is currently unknown.

## Materials and Methods

The current study used data from the following nationally representative data sources:

*(1) South African National Income Dynamics Study (SA-NIDS) (2008–2017)* We used cross-sectional data from the South African National Income Dynamics Study (SA-NIDS) gathered between 2008–2017. Details of these data sources were described elsewhere (NIDS 2018). Survey participants were selected using a systematic random sample in a multistage stratified cluster sampling design setting. The current study only included women 16 years or older who had given birth. The survey participants were chosen through a multistage stratified cluster sampling method. Given the limited representation of other demographic groups, this study focussed on self-identified Black respondents, who comprised 85% of all respondents aged 15 and above. Children’s age, sex, ethnicity, height (cm), weight (kg), any illness at birth, mother/father alive/dead, mothers’ education level, household income, having medical insurance coverage (yes/no), anthropometric measures (weight, height), marital status, parity which was dicotomised using the median < 3 vs 3+, as well as available lifestyle factors including smoking, excess alcohol use and exercise (never vs. at least once a week) were analyzed. Body mass index (BMI) (kg/m^2^) was calculated for each woman. Women were classified as underweight: < 18.5 kg/m^2^, normal weight: 18.5–24.9 kg/m^2^, overweight: 25–29.9 kg/m^2^ and obese: 30+ kg/m^2^.

(2) *General Household Surveys (2008–2021)* Conducted by Statistics South Africa, the General Household Survey (GHS) serves as a crucial data source for South African society, including areas like health, education, labour, and service delivery (General Household Survey 2008–2021). The GHS monitors the progress of development in South Africa and reports service disparities across the country. Briefly, South Africa was divided into 103,576 enumeration areas (EA). The census Enumeration Areas (EAs), supplemented by additional EA information, served as foundational elements in shaping the primary sampling units (PSUs) for the Master Sample. This approach was adopted because EAs encompassed the whole country and contained essential data for stratification and PSU formation. The General Household Survey (GHS) is an annual survey conducted by Statistics South Africa (Stats SA). The GHS was initiated in 2002 and has since become a vital instrument for collecting statistical data on various household topics in South Africa. The GHS is a major data source for health and education to labour and service delivery. The current study utilized a subset of data from pregnant women who participated in the GHS surveys between 2008 and 2021. Women’s age, marital status, education level, household income, and whether they received medical aid were evaluated as potential correlates of food insecurity. Food insecurity was defined by the measure “Skipped meals 5 or more days in the past 30 days” due to a lack of food (Yes/No). This variable was used as a proxy for “food insecurity” and was considered the secondary endpoint.

### Outcome measure

The primary outcome was the stunting among children younger than 5 years of age according to the World Health Organization (WHO) 2007 definition (WHO, 2007). We used children’s age (months) and height (cm) and classified them as stunted if the height-for-age z-score which was calculated as the difference between the observed and the mean of the reference group by the standard deviations calculated for the reference group. Stata 16.0 Macro *zanthro* (Vidmar et al., 2014; Stata, 2019). Children were classified as stunted-if their standardized height-for-age score was less than − 2 standard deviations (SD) of the WHO-Child Growth Standards. Using the same Stata macro, we calculated “weight-for-age”. We classified the children as underweight if their standardized score < − 2 SD of the reference group. The current study excluded those with missing information (< 3%).

### Statistical analysis

In our analysis, the survey populations were described using percentages. We first used the multivariable logistic regression models, which accounted for the multistage survey sampling nature of the design. The second part of the analysis focussed on population-level impacts of low-socioeconomic conditions and maternal anthropometric measures on odds of stunting using a multifactorial version of the epidemiologic measure called population-attributable risk. The goodness of fits of the models was assessed according to the Hosmer–Lemeshow test statistics.

## Results

### South African National Income Dynamic Surveys (SA-NIDS): prevalence and correlates of stunting: 2008–2017

There were 14,151 children under the age of 5 in SA NIDS surveys. Of these, 3,619 (26%) were classified as stunted. The individual and population-level impacts of the risk factors for stunting are detailed in Table 1. In the overall study population, lower household income was significantly associated with increased odds of stunting (aORs ranged from 1.54 (1500–2999 ZAR) to 2.16 (< 1500 ZAR)). Mothers’ marital and education status were also identified as significant correlates of stunting, where single/not cohabiting women and those without education were more likely to have stunted children (aOR: 1.53 and 1.72 respectively). Women who did not have medical aid were more likely to have stunted children compared to those who had medical aid (aOR: 1.92, *p* < 0.001). Children born to mothers who were underweight/shorter than 1.60 cm were also more likely to be stunted (aOR: 1.68, p < 0.001).

**Table 1:** Correlates of stunting among children younger than 5 years of age (n = 14,151) (mother's median age: 27 years, IQR: 22–25)

Characteristics		aOR (95% CI)	*p* value	PAR% (95% CI)
*Child’s sex*				**10% (8%, 12%)**
Female	51%	1		
Male	49%	1.23 (1.14, 1.33)	< 0.001	
*Child’s birth weight*				**7% (5%, 9%)**
≥ 2500 g	89%	1		
< 2500 g	11%	1.91 (1.68, 2.17)	< 0.001	
*Child’s current weight*				**13% (10%, 15%)**
Underweight	12%	1		
Normal weight	62%	1.17 (1.00, 1.36)	0.050	
Overweight	26%	1.64 (1.38, 1.94)	< 0.001	
*Child’s illness at birth*				–
No	97%	1		–
Yes	3%	1.21 (0.98, 1.50)	0.068	–
*Ethnicity*				
Other	14%	1		–
Black	86%	1.05 (0.93, 1.17)	0.430	–
*Mother’s age at birth *				
20+ years old	74%	1		
< 20 years old	26%	1.06 (0.97, 1.15)	0.201	
*Mother/father dead*				**2% (1%, 4%)**
No	96%	1		
Yes	4%	1.43 (1.17, 1.75)	0.001	
*Mother’s marital status*				**31% (26%, 35%)**
Married/cohabiting	18%	1		
Single	82%	1.53 (1.32, 1.79)	< 0.001	
*Mother’s education/tertiary*				**36% (32%, 40%)**
Some education	14%	1		
No education	86%	1.72 (1.51, 1.96)	< 0.001	
*Mother’s employment status*				**14% (11%, 17%)**
Employed	19%	1		
Not employed	81%	1.20 (1.08, 1.33)	< 0.001	
*Household income*				**45% (39%, 0.517)**
No regular income/< 1500 ZAR	83%	2.16 (1.64, 2.85)	< 0.001	
1500 ≤ income < 3000 ZAR	6%	1.87 (1.48, 2.37)	< 0.001	
3000 ≤ income < 4000 ZAR	7%	1.54 (1.17, 2.02)	0.002	
≥ 4000 ZAR	4%	1		
*Do you have a medical insurance coverage?*				**46% (41%, 51%)**
Yes	7%	1		
No	93%	1.92 (1.60, 2.29)	< 0.001	
*Mother smoke*				**1% (0%, 3%)**
No	93%	1		
Yes	7%	1.19 (1.03, 1.38)	0.023	
*Mother’s alcohol intake*				**1% (0%, 3%)**
< 3 per week	99%	1		
3+ per week	1%	1.43 (0.90, 2.30)	0.136	
*Mother: exercise*				**8% (6%, 11%)**
At least once/week	18%	1		
Never	82%	1.11 (1.01, 1.23)	0.048	
*Parity*				**5% (3%, 7%)**
< 3 children	48%	1		
3+ children	52%	1.21 (1.10,1.33)	< 0.001	
*Mother’s weight/height*				**28% (25%, 31%)**
Normal weight^b^/height: ≥ 160 cm	37%	1		
Short: < 160 cm/underweight^a^	63%	1.68 (1.60, 1.77)	< 0.001	
*Mother’s weight/height*				**14% (11%, 15%)**
Normal weight^b^/height: ≥ 150 cm	88%	1		
Short: < 150 cm/underweight^a^	12%	1.91 (1.64, 2.20)	< 0.001	

^a^BMI < 18.5 kg/m^2^

^b^18.5 kg/m^2^ ≤ BMI < 25 kg/m^2^

^c^BMI ≥ 25 kg/m^2^

Other characteristics associated with children: male gender (aOR: 1.23, p < 0.001), low birth weight (aOR: 1.91, < 0.001) and children with dead parent(s) (aOR: 1.43, *p* = 0.001). Mothers’ lifestyle characteristics, including smoking regularly (aOR: 1.19, *p* = 0.023), lack of exercise (aOR: 1.11, *p* = 0.048), higher parity (aOR: 1.21, *p* < 0.001), were also associated with stunting of the offspring. Approximately one-third of the stunted children were associated with single and unmarried women. At the same time, 45% and 46% of the stunting were attributed to children from low-household income and no medical aid, respectively (Table 1).

### Temporal trends in prevalence and population-level impacts of socioeconomic characteristics on stunting: South African National Income Dynamic Surveys (SA-NIDS)

**Fig. 1:**
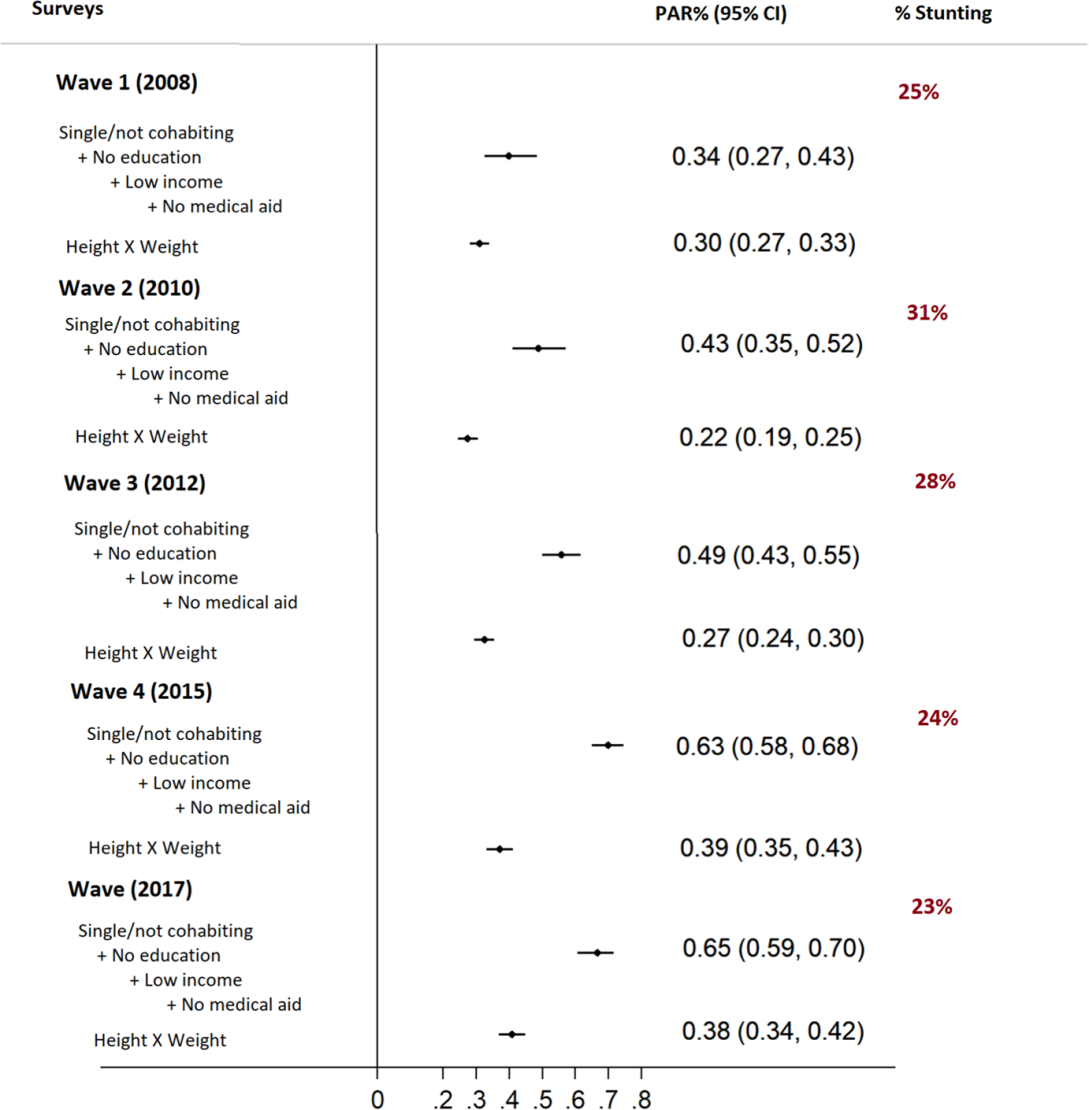
Combined population-level impact of socioeconomic conditions: low income, no education, being single/not cohabiting and not having a medical insurance on stunting children < 5 years old by survey years. Wave 1, wave 2, wave 3, wave 4 and wave 5 are the South African National Income Dynamic Surveys conducted in 2008, 2010, 2012, 2015 and 2017; Height (< 160 cm) × Weight (< 18.5, underweight): mother’s anthropometric measure

Overall, 26% of the children were identified as stunted, which changed from 25% in 2008 to 31% in 2010 and declined to 24% in 2015 and 23% in 2017 (Fig. 1). Consistent with the findings in Table 1, the combined impacts of the four low socioeconomic indicators on stunting increased substantially overtime which ranged from PAR%s: 34% (95% CI 27%, 43%) in 2008, 43% (95% CI 35%, 52%) in 2010, 49% (95% CI 43%, 55%) in 2012, 63% (95% CI 58%, 68%) in 2015 and 65% (95% CI 59%, 70%) and 2017 respectively (Fig. 1). Non-overlapping confidence intervals were interpreted as significant differences across the survey years. Population-level impact of the other characteristics, including male-gender and mothers’ lifestyle characteristics, including smoking, lack of exercise, and higher parity, was less than 15% (ranging from 1 to 13%) due to the low prevalence and low ORs of these characteristics in the population (Table 2).

**Table 2:** Correlates of hunger/food insecurity among pregnant women in General Household Surveys (2008–2021) (N = 22,814)

		2008–2010 (n = 4906)	2011–2013 (n = 6634)	2014–2016 (n = 5916)	2017–2019 (n = 4848)	2020/2021 (n = 510)
Food insecurity		28%	26%	26%	23%	25%

Characteristics	%^a^	Adjusted OR (95% CI)	Adjusted OR (95% CI)	Adjusted OR (95% CI)	Adjusted OR (95% CI)	Adjusted OR (95% CI)
*Age (median, IQR),years*	26 (22–32)	26 (21–31)	26 (21–31)	26 (22–32)	27 (22–32)	27 (22–33)
< 20 years	16%	1.45 (1.23, 1.70)	1.60 (1.35, 1.88)	1.69 (1.33, 2.14)	1.52 (1.23, 1.88)	1.74 (0.93, 3.25)
20–29 years	52%	1.20 (1.07, 1.36)	1.07 (0.95, 1.22)	1.20 (1.01, 1.41)	1.31 (1.13, 1.52)	0.97 (0.63, 1.50)^b^
30+ years	32%	1	1	1	1	1
*Marital status*						
Married	23%	1	1	1	1	1
Single	77%	2.35 (2.00, 2.79)	2.77 (2.37, 3.23)	2.40 (2.04, 2.82)	2.60 (2.13, 3.16)	2.57 (1.43, 4.62)
*Education*						
Primary school or higher	95%	1	1	1	1	1
< Primary school	5%	1.91 (1.38, 2.64)	2.07 (1.60, 2.70)	1.65 (1.17, 2.32)	2.76 (1.75, 4.36)	2.68 1.37, 5.21)
*Income*						
< 1500 ZAR	29%	3.36 (2.82, 4.01)	3.26 (2.81, 2.79)	3.55 (2.92, 4.32)	3.02 (2.52, 3.61)	2.42 (1.29,4.56)
1500–2999 ZAR	29%	2.26 (1.89, 2.70)	2.33 (2.00, 2.70)	2.72 (2.27, 3.25)	2.44 (1.94, 2.76)	2.32 (1.27, 4.24)
3000–3999 ZAR	11%	2.00 (1.57, 2.54)	1.76 (1.45, 2.15)	2.18 (1.73, 2.74)	2.31 (1.94, 2.76)	1.44 (0.83, 2.50)^b^
4000+ ZAR	31%	1	1	1	1	1
*Head of the household*						
Male	49%	1	1	1	1	1
Female	51%	1.52 (1.36, 1.69)	1.57 (1.38, 1.80)	1.53 (1.33, 1.76)	1.60 (1.36, 1.87)	1.41 (1.15, 1.73)
*Do you have a medical insurance coverage?*						
Yes	11%	1	1	1	1	1
No	89%	5.32 (3.88, 7.32)	6.56 (4.78, 9.00)	5.23 (3.88, 7.03)	5.22 (3.30, 8.27)	4.10 (2.57, 6.55)
		Population attributable risk (PAR%) (95% CI)^c^ (individual risk factors)				
Single/not cohabiting	48% (43%, 53%)		55% (52%, 59%)	51% (47%, 55%)	53% (48%, 56%)	55% (50%, 60%)
Low income	42% (38%, 45%)		41% (38%, 44%)	49% (41%, 52%)	40% (36%, 44%)	38% (32%, 45%)
Low education	10% (8%, 13%)		11% (7%, 15%)	9% (6%, 12%)	11% (8%, 15%)	10% (7%, 14%
Female HH head	22% (19%, 26%)		22% (18, 25%)	21% (18%, 23%)	24% (21%, 27%)	19% (16%, 22%)
No medical insurance coverage	79% (76%, 82%)		83% (80%, 86%)	81% (78%, 83%)	80% (77%, 82%)	79% (76%, 80%)
	Population attributable risk (PAR%) (95% CI)^c^ (combined impact of the risk factors)					
Single/not cohabiting + low income + low education + female HH head + no medical aid		87% (85%, 88%)	87% (85%, 89%)	89% (87%, 90%)	83% (79%, 86%)	85% (82%, 88%)

^a^Distribution of the characteristics in combined population

^b^Not significant

^c^Combined impact of all five characteristics on hunger/food insecurity. For example, in 2020/2021, 85% of the hunger/insecurity could have been avoided if it was possible to modify (at least theoretically) single/not cohabiting, low education, female household (HH) head and no medical aid

### Province-specific analysis: population-level impacts of socioeconomic characteristics on stunting: the General Household Survey (GHS)

**Fig. 2:**
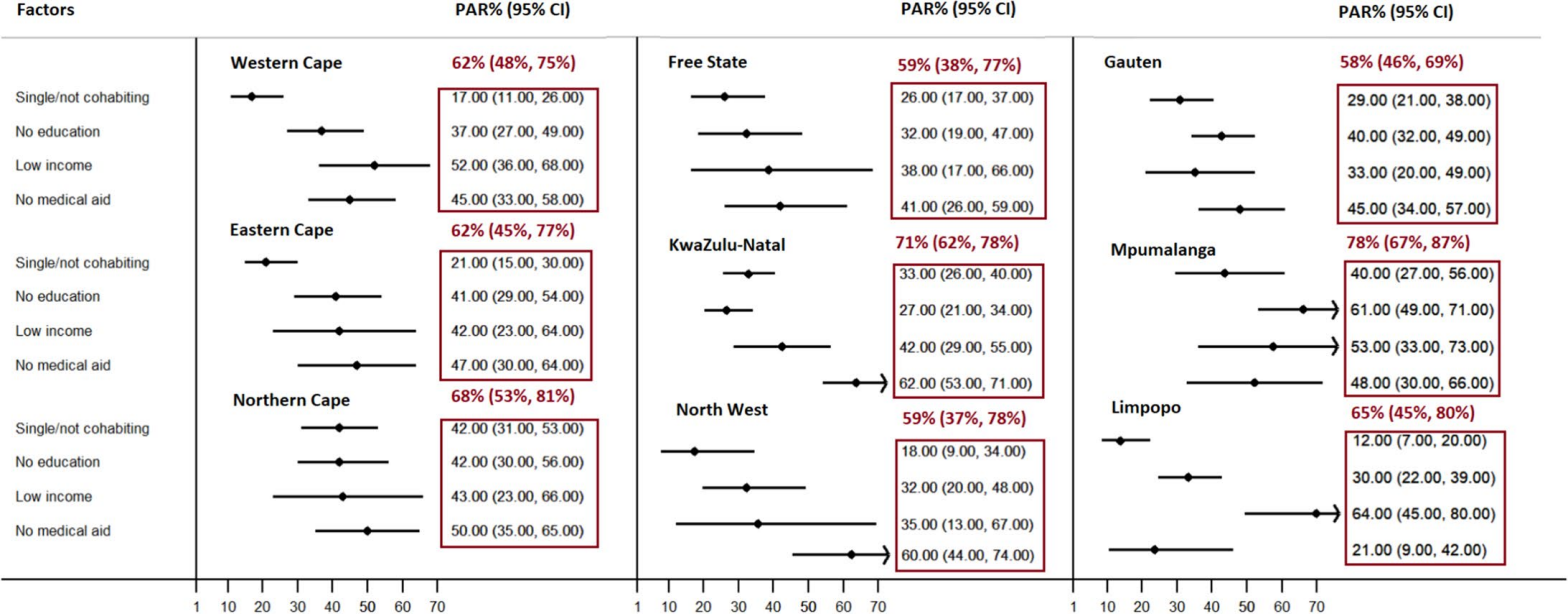
State-specific individual and combined population-level impacts of maternal and house-hold characteristics on stunting: South African General Household Surveys. Province-specific prevalences are: Western Cape (24%), Eastern Cape (26%), Northern Cape (29%), Free State (31%), KwaZulu-Natal (25%), North West (26%), Gauteng (23%), Mpumalanga (19%), Limpopo (31%)

In our province-stratified analysis, we observed significant variations across the provinces where Free State, Limpopo, Free State and Northen Cape had the highest stunting rates at 31% (both Limpopo and Free State) and 29%. Population-level impacts of the low socioeconomic characteristics, including household income, lack of medical aid, maternal marital status, and educational level, significantly contributed to the increased prevalence of stunting across all provinces. More than 60% of the stunted children were collectively attributed to these four characteristics in 6 provinces out of 9. Low household income and the absence of medical aid were identified as the primary risk factors for stunting, with PAR% ranging between 35 and 64% across provinces (Fig. 2).

### Correlates of food insecurity in pregnant women: General Household Surveys: 2008–2021

Data from 22,814 individuals were analyzed. We identified the temporal trends in food insecurity among pregnant women who participated in the General Household Surveys conducted from 2008 to 2021. Overall, low socioeconomic indicators were associated with “food insecurity”. Single women were more than twice as likely as married/cohabiting women to report household hunger in all survey periods (aORs: 2.35–2.77). Consistent with these findings, female-headed households were also more likely to indicate a lack of food and hunger (aORs: 1.52, 1.57, 1.53, 1.60 and 1.41 in 2008–2010 to 2020–2021, respectively). In all survey periods, women with less than primary school education were also consistently associated with food insecurity and hunger (aORs: 1.65–2.76). We observed increased odds of food insecurity with lower household income, where women who reported less than 1500 ZAR were more than three times more likely also to report food insecurity at all time points. Women who indicated not having medical aid were more than four times more likely to report food insecurity. These five characteristics were attributed to > 80% of women’s food insecurity/hunger.

## Discussion

Consistent with the global trends, we observed a modest decline in stunting prevalence from 25% in 2008 to 23% in 2017, with significant geographical-level variations across the country. The prevalence of stunting remained as high as 28% in three of the nine provinces: Northern Cape, Free State and Limpopo. According to the United Nations Children’s Fund (UNICEF, 2019) 2019 estimates, 27% of children under five are stunted (WHO, 2020). These unacceptably high rates are alarming and substantially higher than the targets set by the *Sustainable Development Goals* 2012–2025 global action plan to reduce the stunting by 40% in 2025 compared to the baseline year 2010 (UNICEF Transforming our World 2015; UNICEF From the first hour of life, 2016).

In our study, low-income households without medical aid were identified as the most influential risk factors associated with more than 45% of stunted children. Mothers’ marital and education status also significantly impacted the increasing prevalence of stunting, where 31% and 36% of the cases were attributed to single women and no education, which were previously reported (Black et al., 2008; Frongillo et al., 1997). The combined impact of these four characteristics on stunting increased substantially over time and was collectively associated with 65% of stunted children in 2017. Although, most of these results are aligned with previous research, the population-level impact of household and maternal characteristics is exclusively unique to the current study population. Intuitively, if it were possible to modify these characteristics (at least theoretically), (65%*3619) = 2,352 of the stunted children would have been prevented, which would have reduced the overall prevalence of stunting from 26% (3619/14,151) to 9% [(3619–2352)/14,151].

Prolonged exposure to poverty, often driven by low socioeconomic conditions, has been linked to child malnutrition and growth failures, including stunting in other populations (Victora et al., 2008). Low-socioeconomic conditions also limits access to clean water and sanitation which can lead to diarrheal diseases and worsen malnutrition (Black et al., 2008; Case & Menendez, 2007; Frongillo et al., 1997). Moreover, poverty is also one of the significant barriers to accessing healthcare services, which can hinder the early detection and treatment of malnutrition and related health issues (Richter et al., 2012).

One of the most remarkable results from our study was the strong association between the mothers’ anthropometric measures and stunting. Approximately one-third of the stunted children were associated with smaller-stature women, height < 160 cm/underweight. We identified 160 cm as the most influential threshold height among underweight women on the increased prevalence of stunting (PAR%: 28%), compared to those < 150 cm and underweight (PAR%: 14%). At a population level, 14% of the stunted children were associated with women < 150 cm/underweight women, which was doubled to 28% for women < 160 cm/underweight. This difference was primarily due to the low prevalence of women < 150 cm, compared to those < 160 cm (10% vs 52% respectively). Although these results are broadly consistent with previous research, our findings are exclusively unique to South African women and their off-spring which identified 160 cm/underweight as to be the important threshold compared to those 150 cm/underweight as widely reported in other populations (Özaltin et al., 2010; Subramanian et al., 2009; Addo et al., 2013; Perumal et al., 2018; Subramanian et al., 2009).

As reported previously, malnutrition which is strongly linked to poverty is an established risk factor for childhood growth failure, including stunting (Martorell & Zongrone, 2012). Despite the accelerated global actions to reduce poverty, our study provided compelling evidence for high rates of household hunger which remained broadly stable over time with little improvement. More than 20% of the households reported food insecurity in South Africa, compared to the global average of 8.9% (WHO-UNICEF, 2019). The impact of the pandemic on food insecurity was relatively modest due to the government grants, which were more than doubled in 2021/2022 compared to 2020 (44% and 20%, respectively) (Wand et al., 2023). These estimates provide strong evidence for the high burden of poverty in South Africa. As of 2022, the country is also substantially behind *the Sustainable Development Goals* set by the world leaders, which aimed to “end hunger, achieve food security and improved nutrition and promote sustainable agriculture” by 2030.

### Limitations

This is a cross-sectional study and has the same limitations associated with this design. We only included Black South Africans due to the low response rates from the other ethnicities, as they were excluded from the analysis (< 15%). Although the study collected anthropometric characteristics of the mothers and children, personal, behavioural and sociodemographic characteristics were self-reported by the survey participants. Nevertheless, the survey data provided a unique opportunity to examine the population-level influences of socioeconomic conditions, behavioural factors, and stunting within a large cohort of children under 5. The connection between food insecurity and stunting, our study utilized different datasets to explore these variables. While they were not analyzed within the same dataset, our approach involved a comprehensive review of existing data to draw correlations between low socioeconomic conditions (a broader category that includes aspects of food insecurity) and stunting. Finally, a population-level impact of risk factors requires an assumption of causality, which our data cannot confirm. However, many risk factors identified as significant predictors of stunting have previously been recognized as established risk factors.

## Conclusion

Despite a modest decline, our study reported high stunting rates among South African children. Our findings reinforce the widely recognized relationship between socioeconomic factors and child malnutrition. Investigating the temporal trends in stunting rates is critical for evaluating the current prevention and care models, which are currently lacking in South Africa. Quantifying the burden of stunting has significant clinical and public health implications and may provide guidance for future prevention efforts. The most compelling finding from our study was that South Africa is substantially behind the global targets set for hunger and stunting. Our findings suggest that despite some improvements in stunting rates between 2008 and 2017, South Africa still has one of the world’s highest rates of stunted children. These estimates are behind the projected goals set by UNICEF 2030. Most importantly, besides mothers’ anthropometric measures, the distribution and population-level impacts of the low-socioeconomic characteristics remain as the most influential risk factors across the provinces. Given the current state of HIV in the country, a cost-effective prevention strategy may include multi-component prevention care models by incorporating infant feeding practices and malnutrition screening/treatment programs into current HIV testing/treatment programs whenever possible. Our findings suggest that merging these two programs could create multi-component prevention care models to reduce child malnutrition. In addition to raising awareness of safe sexual behaviors, these care models should emphasize the benefits of breastfeeding exclusively for the first six months of life and continuing breastfeeding up to two years of age or beyond, as recommended by WHO guidelines. Additionally, they should promote malnutrition screening programs.

### Supplementary Information

Below is the link to the electronic supplementary material.

Supplementary file1 (DOCX 25 kb)

Supplementary file2 (DOCX 14 kb)

## Data Availability

South African National Income Dynamics Study (SA-NIDS): Data are available in a public, open access repository. The dataset is available at https://www.datafirst.uct.ac.za/. *Demographic and Health Survey Program*: Data are available in a public, open access repository. The dataset is available at https://dhsprogram.com/data/available-datasets.cfm

## Code Availability

Appendix presented the codes generated in SAS-Software to estimate PAR%.
