# Supplementary material for: Preventing Stunting in South African Children Under 5: Evaluating the Combined Impacts of Maternal Characteristics and Low Socioeconomic Conditions
Source: J Prev (2022). 2024 Feb 28;45(3):339–55. doi: 10.1007/s10935-024-00766-2 (PMC11033229; doi:10.1007/s10935-024-00766-2)
Supplement: Supplementary file 1 — Supplementary file1 (DOCX 25 kb) [file 10935_2024_766_MOESM1_ESM.docx]

**Supplementary File:** Study populations

1. *South African National Income Dynamics Study (SA-NIDS) (2008-2017)*:

SA-NIDS employs a stratified two-stage cluster sampling design, leveraging the latest national census data for its sampling frame. The country is divided into strata based on geographic and socio-economic criteria such as provincial divisions and urban-rural distinctions. This stratification ensures that the sample is representative of South Africa's diverse socio-economic landscape. In the first stage, primary sampling units (PSUs) are selected randomly but in a stratified manner from different regions and community types. These PSUs typically consist of geographic areas or enumeration areas (EAs). In the second stage, households within these PSUs are chosen through systematic random sampling, giving each household in the PSU an equal chance of selection. The SA-NIDS is designed as a panel study, meaning it repeatedly surveys the same households over time. This allows for the collection of longitudinal data, shedding light on changes in socio-economic conditions. To accurately represent the national population, the data are carefully weighted. This weighting adjusts for the probability of selection and non-response, ensuring the survey results reflect the demographic makeup of the entire population. The methodology used in SA-NIDS is crucial for understanding the dynamics of income, poverty, and social mobility within South Africa over an extended period.

We used cross-sectional data from the South African National Income Dynamics Study (SA-NIDS). Details of these data sources were described elsewhere (NIDS 2018). Briefly, there were 32,686 (41%) men and 47,584 (59%) women who participated in one of the five NIDS surveys during the period of 2008-2017. The current study only included data from children aged younger than 5 years (n=14,151) and their mothers.

1. The South African General Household Survey (GHS) (2008-2021):

The South African General Household Survey employs a complex, multistage sampling design to ensure that the findings are representative of the national population. The process begins with the development of a sampling frame, typically based on the latest population census data. This frame divides the country into various enumeration areas (EAs), which are then stratified based on geographical and demographic characteristics such as province, urban or rural status, and other relevant factors. This stratification is crucial for capturing the diversity of the South African population and for ensuring that all significant segments are included in the sample. In the initial stage of sampling, primary sampling units (PSUs), usually these EAs, are selected from each stratum through systematic random sampling. In the subsequent stage, households within these selected EAs are randomly chosen for participation in the survey. To correct for any over- or under-sampling in certain areas or demographic groups, the data collected are weighted. This weighting process adjusts for the varying probabilities of selection among different segments of the population, ensuring that the survey results are accurately representative of the entire population.

Among the participants in the five GHS surveys conducted from 2008 to 2021, there were 511,481 men (47%) and 574,045 women (53%). The current study exclusively included data from 22,814 pregnant women.
