# Supplementary material for: Preventing Stunting in South African Children Under 5: Evaluating the Combined Impacts of Maternal Characteristics and Low Socioeconomic Conditions
Source: J Prev (2022). 2024 Feb 28;45(3):339–55. doi: 10.1007/s10935-024-00766-2 (PMC11033229; doi:10.1007/s10935-024-00766-2)
Supplement: Supplementary file 2 — Supplementary file2 (DOCX 14 kb) [file 10935_2024_766_MOESM2_ESM.docx]

**Appendix. Development of Population Attributable Risk percentage (PAR%)**

In order to estimate the population-level contribution of mothers’ anthropometric measurements and socioeconomic conditions, we estimated the population attributable risk$(PAR\%)$ in multifactorial setting after accounting for the correlation structure of the risk factors (Wand et al., 2022). The $PAR\%$ was calculated as a bivariate function of the prevalence of an exposure ($p$) and its measure of association with primary outcome. For a binary outcome variable, i.e. stunted vs. not stunted (1/0):

$PAR\%$

$$=\frac{p(OR-1)}{p(OR-1)+1}=1-\frac{1}{\sum_{s=1}^{2} p_{s}OR_{s}}$$

$s$ = levels of a risk factor. In multifactorial setting:

$PAR\%$

$$=\frac{\sum_{s=1}^{S} p_{s}(OR_{s}-1)}{1+\sum_{s=1}^{S} p_{s}(OR_{s}-1)+1}=1-\frac{1}{\sum_{s=1}^{S} p_{s}OR_{s}}$$

Where $OR_{s}$and $p_{s}$, $s=1,...,S$. Multinomial probabilities were calculated at each unique level combination of risk factor(s) and their respective odds ratios.

SAS 9.0 (SAS Institute Inc., Cary, NC,USA)

**Sample SAS code for calculating the *PAR%***

Please see publicly available SAS Macro:

<https://www.hsph.harvard.edu/donna-spiegelman/software/par/>

**Sample SAS code for calculating the POPULATION ATTRIBUTABLE RISK**

**Program:**

**title 'make variance-covariance matrix of beta coefficients';**

**PROC** **LOGISTIC** DATA=TEMP1 COVOUT OUTEST=DATA.BETA;

MODEL **stunted** (EVENT="1")=

Single_mother

No_medical_Aid

Low_income

Mother_antropometric

/RL;

**RUN**;

**title 'make dataset of joint prevalences of modifiable and un-modifiable risk factors';**

**PROC** **SORT** DATA=all; By

Single_mother

No_medical_Aid

Low_income

Mother_antropometric

;

**run**;

**PROC** **MEANS** NOPRINT DATA=all ; VAR id;

OUTPUT OUT= PREV N=fq;

By

Single_mother

No_medical_Aid

Low_income

Mother_antropometric

;

**run**;

%***hwpar***(bdata= BETA,

pdata= PREV,

n_or_p=n,

n_or_pname=FQ,

**MODVAR** **(MODIFIABLE RISK FACTORS)**=

Single_mother

No_medical_Aid

Low_income

Mother_antropometric

,

**FIXEDVAR (NON MODIFIABLE RISK FACTORS)**=

Age

);

Output:

**[[[[[[[[[[[[[[[[[[[[[[[[[[[[[[[[[[[[[[[[[[[[[[[[[[[[[[[[[[[[[[[[[[[[[[[[[[[[[[[[[[[[[[[[[[[[[[[[[[[[[[[[[[[[[[[[[[[[[[[[[[[[[[[[[[**

The SAS System **13**:**37** Wednesday, AUGUST **23**, **2023** **1**

**Partial PAR (95% CI) for**

**modifiable vbls:**

Single_mother

No_medical_Aid

Low_income

Mother_antropometric

**fixed vbls :** Age

**0.65**  (**0.59**, **0.70)**

**[[[[[[[[[[[[[[[[[[[[[[[[[[[[[[[[[[[[[[[[[[[[[[[[[[[[[[[[[[[[[[[[[[[[[[[[[[[[[[[[[[[[[[[[[[[[[[[[[[[[[[[[[[[[[[[[[[[[[[[[[[[[[[[[[[**

Wand, H. & Ramjee, G. (2012) Combined impact of sexual risk behaviors for HIV seroconversion among women in Durban, South Africa: implications for prevention policy and planning. *AIDS Behav.* 15(2):479–86.
